# In vitro dose effect relationships of actinium-225- and lutetium-177-labeled PSMA-I&T

**DOI:** 10.1007/s00259-022-05821-w

**Published:** 2022-05-12

**Authors:** Eline A. M. Ruigrok, Giulia Tamborino, Erik de Blois, Stefan J. Roobol, Nicole Verkaik, Marijke De Saint-Hubert, Mark W. Konijnenberg, Wytske M. van Weerden, Marion de Jong, Julie Nonnekens

**Affiliations:** 1 Department of Radiology and Nuclear Medicine, Erasmus MC Cancer Institute, Erasmus University Medical Center, 3000 CA Rotterdam, The Netherlands; 2 Department of Experimental Urology, Erasmus University Medical Center, 3000 CA Rotterdam, The Netherlands; 3 Research in Dosimetric Application, Belgian Nuclear Research Centre (SCK CEN), Mol, Belgium; 4 Department of Molecular Genetics, Erasmus MC Cancer Institute, Erasmus University Medical Center, 3000 CA Rotterdam, The Netherlands

**Keywords:** Prostate-specific membrane antigen, Radioligand therapy, Prostate cancer, Relative biological effectiveness, Actinium-225, Lutetium-177

## Abstract

**Purpose:**

Targeting the prostate-specific membrane antigen (PSMA) using lutetium-177-labeled PSMA-specific tracers has become a very promising novel therapy option for prostate cancer (PCa). The efficacy of this therapy might be further improved by replacing the β-emitting lutetium-177 with the *α*-emitting actinium-225. Actinium-225 is thought to have a higher therapeutic efficacy due to the high linear energy transfer (LET) of the emitted *α*-particles, which can increase the amount and complexity of the therapy induced DNA double strand breaks (DSBs). Here we evaluated the relative biological effectiveness of [^225^Ac]Ac-PSMA-I&T and [^177^Lu]Lu-PSMA-I&T by assessing in vitro binding characteristics, dosimetry, and therapeutic efficacy.

**Methods and results:**

The PSMA-expressing PCa cell line PC3-PIP was used for all in vitro assays. First, binding and displacement assays were performed, which revealed similar binding characteristics between [^225^Ac]Ac-PSMA-I&T and [^177^Lu]Lu-PSMA-I&T. Next, the assessment of the number of 53BP1 foci, a marker for the number of DNA double strand breaks (DSBs), showed that cells treated with [^225^Ac]Ac-PSMA-I&T had slower DSB repair kinetics compared to cells treated with [^177^Lu]Lu-PSMA-I&T. Additionally, clonogenic survival assays showed that specific targeting with [^225^Ac]Ac-PSMA-I&T and [^177^Lu]Lu-PSMA-I&T caused a dose-dependent decrease in survival. Lastly, after dosimetric assessment, the relative biological effectiveness (RBE) of [^225^Ac]Ac-PSMA-I&T was found to be 4.2 times higher compared to [^177^Lu]Lu-PSMA-I&T.

**Conclusion:**

We found that labeling of PSMA-I&T with lutetium-177 or actinium-225 resulted in similar in vitro binding characteristics, indicating that the distinct biological effects observed in this study are not caused by a difference in uptake of the two tracers. The slower repair kinetics of [^225^Ac]Ac-PSMA-I&T compared to [^177^Lu]Lu-PSMA-I&T correlates to the assumption that irradiation with actinium-225 causes more complex, more difficult to repair DSBs compared to lutetium-177 irradiation. Furthermore, the higher RBE of [^225^Ac]Ac-PSMA-I&T compared to [^177^Lu]Lu-PSMA-I&T underlines the therapeutic potential for the treatment of PCa.

**Supplementary Information:**

The online version contains supplementary material available at 10.1007/s00259-022-05821-w.

## Introduction

Over the last decade, prostate-specific membrane antigen (PSMA) radioligand therapy (RLT) has become a major focus point in the treatment of advanced prostate cancer (PCa), such as for treatment of patients with (metastasized) castration resistant prostate cancer (CRPC). Over 90% of PCa patients show an overexpression of the membrane bound PSMA, making it an ideal protein to target during RLT [1–3]. During PSMA-RLT, PSMA-specific tracers, such as the small molecule inhibitors PSMA-I&T and PSMA-617, are labeled with a radionuclide that emits ionizing radiation particles, inducing DNA damage leading to cell death. Our previous work has shown that PSMA-I&T and PSMA-617 have comparable in vitro cell-binding characteristics and in vivo tumor uptake [4].

In current clinical trials, lutetium-177 (β-emitter) is most often the radionuclide of choice, showing success rates in up to 80% of the treated patients, based on decline in prostate-specific antigen (PSA) blood levels [3, 5]. In a recently published clinical phase 3 study, metastatic CRPC patients were treated with [^177^Lu]Lu-PSMA-617 plus standard care and showed an increased survival of 4–5 months compared to patients receiving standard care alone [6]. Although these results are encouraging, there is a great interest in increasing the therapeutic efficacy of PSMA-RLT to further elongate the survival time, or even cure the patients.

To further increase the therapeutic efficacy of PSMA-RLT, *α*-emitting radionuclides are being explored (pre)clinically to use in addition or instead of lutetium-177. *α*-particles have a higher linear energy transfer (LET) compared to β-particles, which can cause induction of complex DNA double stranded breaks (DSBs) or multiple DSBs in close proximity of each other [7]. Moreover, the range of *α*-particles is shorter compared to β-particles, which may reduce chance of damaging surrounding healthy cells [8]. Currently, there are various *α*-emitting radionuclides that are being explored for PSMA targeted alpha therapy (TAT), such astatine-211, lead-212, bismuth-213, actinium-225, and thorium-227 [9–13]. Of these *α*-emitting radionuclides, actinium-225 is used most often for PSMA-TAT, because of its favorable half-life (9.92 days) and its emission of a total of four *α*-particles in the decay cascade [14].

Preclinical in vivo studies using actinium-225-labeled PSMA-targeting tracers (PSMA-617, RPS-074) so far showed impressive results such as complete tumor response of human PCa xenografts as well as prevention of metastasis formation resulting in significant increase in survival of the mice[9, 15].

The first clinical studies have successfully applied actinium-225-labeled PSMA-I&T and PSMA-617 showing significant decrease in tumor burden, even after limited results in the same patients with the same tracers labeled with lutetium-177 [16–18]. However, PSMA-TAT has shown not to be effective for all included patients. For example, 15% of the 73 included metastatic CRPC PCa patients that were treated with multiple cycles of [^225^Ac]Ac-PSMA-617 showed increased PSA levels after treatment [19].

Furthermore, the use of actinium-225 comes with significant concerns as it can induce more cellular damage compared to lutetium-177, resulting in increased toxicity in healthy PSMA-expressing salivary glands and kidneys. Nevertheless, PSMA-RLT research almost directly moved towards clinical trials, while preclinical evaluations to assess therapeutic efficacy as well as safety, are still relatively limited [1, 4]. To define the full potential of actinium-225 PSMA-TAT, it is essential to first evaluate its relative biological effectiveness compared to the same tracers labeled with lutetium-177. In this study, we have therefore evaluated in vitro binding characteristics, dosimetry, and therapeutic efficacy of [^225^Ac]Ac-PSMA-I&T and [^177^Lu]Lu-PSMA-I&T.

## Material and methods

### Reagents and chemicals

All reagents and chemicals were purchased from Sigma-Aldrich unless specified below.

### Radiolabeling

#### Actinium-225

The labeling of PSMA-I&T with actinium-225 has been described previously [20]. Briefly, PSMA-I&T (Huayi Isotopes Co. via ATT Scintomics) was labeled with actinium-225 (JRC, Karlsruhe, Germany) with a molar activity of 0.225 MBq/nmol. To prevent radiolysis, quenchers ascorbate (1 M) and ethanol (6%), were used [20]. Quality control was performed by assessing radiochemical yield (RCY) by instant thin-layer chromatography and radiochemical purity (RCP) by high-pressure liquid chromatography. Both RCY and RCP were assessed based on measuring francium-221, the daughter of actinium-225. For all labelings, the RCY was >95% and the RCP was >90%.

To make sure the measured radioactivity of francium-221 directly corresponds to the amount of activity of actinium-225, there was at least 30 min between the preparation of the samples and the measurements (during quality control and all experiments), so that an equilibrium had formed between francium-221 and actinium-225 [20].

#### Lutetium-177

The labeling of PSMA-I&T with lutetium-177 was performed as previously described [21, 22]. Briefly, PSMA-I&T was labeled with lutetium-177 (LuMark, IDB Holland) with a molar activity of 40 MBq/nmol. To prevent radiolysis, quenchers were added (3.5 mM ascorbic acid, 3.5 mM gentisic acid, 10 mM methionine). To reach a lower molar activity of 0.225 MBq/nmol for the uptake experiments, unlabeled PSMA-I&T was added to the solution after labeling. For all labelings, the RCY was > 95% and the RCP was > 90%.

### Cell culture

In vitro experiments were performed using the PCa cell line PC3-PIP, kindly provided by prof. Anna Orlova, Uppsala University. PC3-PIP is a PSMA-transfected variant of the non-PSMA expressing PC3 cell line [23]. Cells were grown in RPMI 1640, Glutamax medium (Gibco) supplemented with 10% fetal bovine serum (Gibco), penicillin (100 units/mL) (Gibco), and streptomycin (100 μg/mL) (Gibco) at 37°C and 5% CO_2_. Every other week, 10 μg/mL puromycin (InvivoGen) was added to the medium to ensure stable PSMA-expression. Cells used for experiments were cultured in puromycin-free medium for at least 48 h before the experiment.

### Uptake, IC_50_ displacement, and cellular excretion assay

One day prior to experiments, 750,000 PC3-PIP cells per well were seeded in 6-well plates. The next day, adherent cells were incubated in 1.5 mL culture medium with [^225^Ac]Ac-PSMA-I&T (10^-9^ M, 0.225 kBq/mL) or [^177^Lu]Lu-PSMA-I&T (10^-9^ M, 0.225 kBq/mL) (0.225 MBq/nmol for both) for 1 and 3 h at 37°C and 5% CO_2_. After incubation, cells were washed with phosphate-buffered saline (PBS) (Gibco) twice before lysis with 0.1 M NaOH. Cell lysates were collected and radioactivity was measured using a γ-counter (1480 WIZARD automatic γ counter; PerkinElmer) alongside 100 μL of the radioactive medium as a standard. Uptake was expressed as a percentage of the added activity per 100,000 cells (%AA/100,000 cells). The total number of cells in an additional well was counted for normalization using the countess automated cell counter (Invitrogen). To determine the cellular excretion rate, cells were incubated for 3h with [^177^Lu]Lu-PSMA-I&T (10^-9^ M 40 kBq/mL) (40 MBq/nmol) as described above and washed twice before receiving fresh medium. Medium and cell activity fractions were measured at 0h, 4h, 24h, 48h, and 120 h after incubation.

To determine the IC_50_ ( the concentration of unlabeled PSMA-I&T that needs to be added to block 50% of the maximum uptake of radiolabeled PSMA-I&T) displacement assays were performed. PC3-PIP cells were incubated for 3 h with 1.5 mL of either [^225^Ac]Ac-PSMA-I&T (10^-9^ M, 0.225 kBq/mL) (0.225 MBq/nmol) or [^177^Lu]Lu-PSMA-I&T (10^-9^ M, 40 kBq/mL)(40 MBq/nmol), together with increasing concentrations (10^−13^M – 10^−6^M) of unlabeled PSMA-I&T, whereafter the bound radioactivity was measured as described above.

All uptake and IC_50_ experiments were performed as three independent experiments in triplicate. The cellular excretion assay was performed as two independent experiments in triplicate.

### DSB assay

PC3-PIP cells were seeded onto coverslips in 6 well plates one day prior to the experiment. Cells were treated for 3h in 1.5 mL culture medium with either 0.37 kBq/mL [^225^Ac]Ac-PSMA-I&T or 0.4 MBq/mL [^177^Lu]Lu-PSMA-I&T at 37°C and 5% CO_2,_ whereafter the radioactive medium was replaced with standard culture medium and the cells were placed back in the incubator at 37° C and 5% CO_2_ until fixation. Non-treated cells were taken along as control. Moreover, cells treated with the same radioactive incubation medium in which a ×1000 higher concentration of unlabeled PSMA-I&T were included as block controls for the 0–24h time points. At different time points after incubation (0h, 2h, 16h, 24h, 48h, 72h, and 96h), cells were washed with PBS and fixed with 2% paraformaldehyde in PBS for 15 min at room temperature (RT). Next, cells were stained for DSB marker p53-binding protein 1 (53BP1). The coverslips were washed with PBS, then washed twice for 10 min with PBS + 0.1% Triton X-100 (Merck), and once with PBS+ (100 mL PBS + 0.5 g BSA + 0.15 g glycine). Subsequently, cells were incubated with the primary antibody anti-53BP1 (1:1000, rabbit, Novus Biologicals NB100-904 Lot I) in PBS+ for 90 min at RT. Hereafter, cells were washed with PBS + 0.1% Triton X-100 and incubated with the secondary antibody, goat-anti-rabbit Alexa Fluor 594 (1:1000, Thermo Fisher Scientific) in PBS+ for 60 min at RT. The coverslips were then washed with PBS + 0.1% Triton X-100 and mounted with Vectashield + DAPI (Vectorlabs). Fluorescent imaging was performed with a SP5 confocal microscope (Leica) using z-stack acquisition (×63 oil lens, ×2 zoom). The experiment was performed as two independent experiments and of each coverslip (1 per condition), at least 5 fields of view were imaged. Each image contained 20–50 cells so in total, 100–250 cells were counted for each condition. The images were automatically analyzed using a custom-made macro in ImageJ. Briefly, the z-stacks were compressed to the maximum projection whereafter cell nuclei were automatically segmented into regions of interest (ROIs) based on the DAPI staining. Next, the mean and standard deviation of the 53BP1 signal within a nucleus determined an arbitrary threshold value which was used for 53BP1-foci segmentation followed by counting of the segmented foci. Using the output of the macro, the mean number of 53BP1 foci per cell was determined. Data are shown per experiment in the main and supplemental figure.

### Clonogenic assay

Cells were treated for 3 h in suspension in 2 mL Eppendorf tubes, by placing 100,000 cells in 1 mL of 20 mM Hepes buffered culture medium with increasing concentrations of [^225^Ac]Ac-PSMA-I&T ( 0–0.0037–0.1–0.185–0.25–0.37–0.5–0.75–1.25–1.85–3.7 kBq/mL, 0.225 MBq/nmol) or [^177^Lu]Lu-PSMA-I&T ( 0–0.1–0.2–0.3–0.4–0.5–1–2– MBq/mL,40 MBq/nmol). Extra tubes of 0.37 kBq/mL of [^225^Ac]Ac-PSMA-I&T and 0.4 MBq/mL [^177^Lu]Lu-PSMA-I&T were taken along in which ×1000 higher concentration of unlabeled PSMA-I&T was added as a block. Tubes were incubated at 37°C under rotation. After incubation, the cell suspension was diluted to 450 cells per 3mL for each condition and were seeded in triplicate in 6-well plates and set to grow colonies for 7 days. Next, plates were washed with PBS and colonies were stained with 0.1% Coomassie brilliant blue staining solution (50% methanol, 7% acetic acid, 43% water, 0.1% Coomassie (Thermo Fisher)) at 30 min at RT. Colonies were automatically counted using the gelcounter (Oxford optronix) and results were normalized to non-treated controls.

Experiments were performed as three independent experiments in triplicate. The survival fraction was plotted as a function of absorbed dose to the nucleus and fitted to linear-exponential curves, to generate the dose-response curves. The corresponding standard deviations were calculated assuming a Poisson distribution [24].

### Dosimetry

Dosimetry was performed to calculate the total absorbed dose to the nucleus of the cells during the clonogenic assay. A schematic overview of the performed dosimetry, including all used formulas, can be found in the supplemental material.

We differentiated between the different incubation period with the radioactive medium and the colony growth period. During the incubation period, cells were in suspension and assumed to be spherical. An automatic circle detection algorithm based on Hough transform [25, 26] was used to identify spherical cellular shapes within brightfield confocal microscope images of floating PC3-PIP cells (> 100 cells) and provide as output the radii of each cell (R_C_), from which average, minimum and maximum dimensions were drawn (Table 1). To determine the size of the nucleus, fluorescent confocal microscope images from the DNA-damage assay were used to determine the minimum, average, and maximum nuclear semi-axis dimensions (a, b), assuming an ellipsoidal shape. The nucleus maximum thickness (referred to as h or 2x c, see Table 1 section: attached cells - ellipsoid ) was kept constant at either 3 μm, 4 μm, or 5 μm for minimum, average, and maximum volume respectively, based on previous imaging data analysis. From these dimensions, an equivalent spherical volume was determined for the nucleus of the floating cells. The thickness of the membrane corresponds to an additional radius of 7.5 nm [27, 28].

**Table 1:**
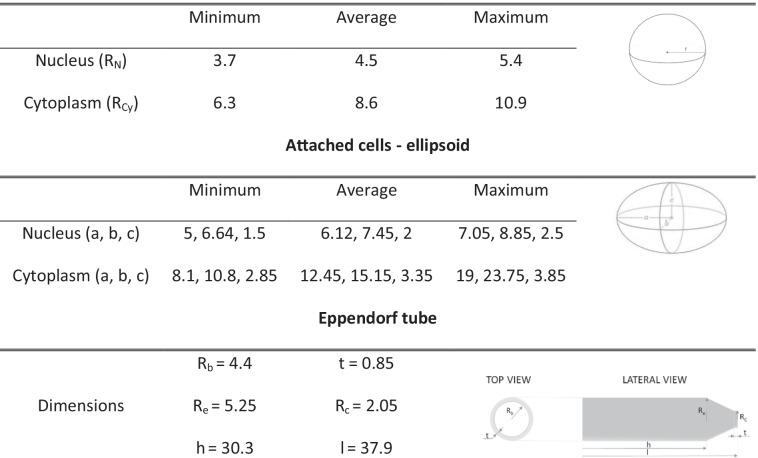
Simulation set-up. Cell and Eppendorf tube dimensions are reported in μm and mm, respectively

The cross dose (cross dose from neighboring/surrounding cells to the cell we are observing) corresponding to lutetium-177 was evaluated with MIRDcell [29] assuming randomly placed spheres (*R*_C_ = 8 μm, R_N_ = 4 μm) within a cubic volume of 500 μm side, with an occupational density experimentally determined ($\rho =\frac{V_{\mathrm{CELLS}}}{V_{\mathrm{MEDIUM}}}=$0. 027%). The cross absorbed dose for actinium-225 was neglected due to its short-range emissions of the *α*-particles with respect to the distance between cells and the low probability of the γ-particles to reach the cellular nucleus.

The non-specific absorbed dose delivered from the medium to the nuclei during incubation was calculated assuming homogeneously distributed radioactivity (100% of the added activity) over a 1 mL volume inside the Eppendorf tube (Table 1). The absorbed dose was scored over the 1 mL volume, as the tubes were constantly in motion. The cross dose during the colony growth period was neglected as the cells were too far away from each other.

The attached cells were assumed to have an ellipsoidal shape, characterized by the same nuclear dimensions determined for the floating cells. The size of the cytoplasm was obtained by arbitrarily increasing the semi axes dimensions, while preserving the a/b ratio (Table 1), fixing the minimum cytoplasm thickness and preserving the volume with respect to the spherical equivalent (< 1 %).

Simulations were performed with the Geant4 Toolkit version 10.03 (6) using the pre-defined ion particle source (ENSDF). The materials defined for the cell compartments are the same as previously published [30]. Electromagnetic and hadronic interactions were simulated with the Livermore and the FTFP_BERT physics lists, respectively. The cut-off value for the production of secondary particles was set to 0.1 μm and the maximum step size in the nucleus was limited to 0.5 nm for *α*-particles. For [^177^Lu]Lu-PSMA-I&T, 1,000,000 particles were stimulated, and for [^225^Ac]Ac-PSMA-I&T 100,000 particles were stimulated to reach a statistical error below 0.5% (error was determined using Monte Carlo approach [31]).

For the time-integrated activity calculations, the full decay of lutetium-177 and actinium-225 was included in the calculations (thus including photons, IC-electrons, auger particles etc.). An instant uptake with a cellular bound fraction of activity corresponding to the average uptake data (i.e. the average uptake between 1 and 3h divided by the number of cells) was assumed. A constant ratio between the membrane bound fraction and the internalized fraction of PSMA-I&T, corresponding to 0.76, was used for both radionuclides [4]. The distribution of the membrane bound fraction and the internalized fraction in the cytoplasm were considered homogeneous. The measured cellular excretion data was fitted and used to evaluate the time-integrated activity after incubation time. The activity was cumulated up to 7 days, in order to obtain the cumulated absorbed dose corresponding to the clonogenic survival assay. The highest activity concentrations were not included in the dose-response correlations, as further explained in the discussion section.

### Statistical analysis

All graphs and statistical analysis were created using the Graphpad Prism software (version 6.01). Significant differences were evaluated using a one-way ANOVA test, *p* values below 0.05 were considered significant. IC_50_ values were determined by plotting (non-linear regression) IC_50_ curves on normalized data. Fitting was performed according to the least square method, with Pearson *R*^2^ as parameter for its correctness.

## Results

### [^225^Ac]Ac-PSMA-I&T and [^177^Lu]Lu-PSMA-I&T have similar in vitro binding characteristics

**Fig. 1:**
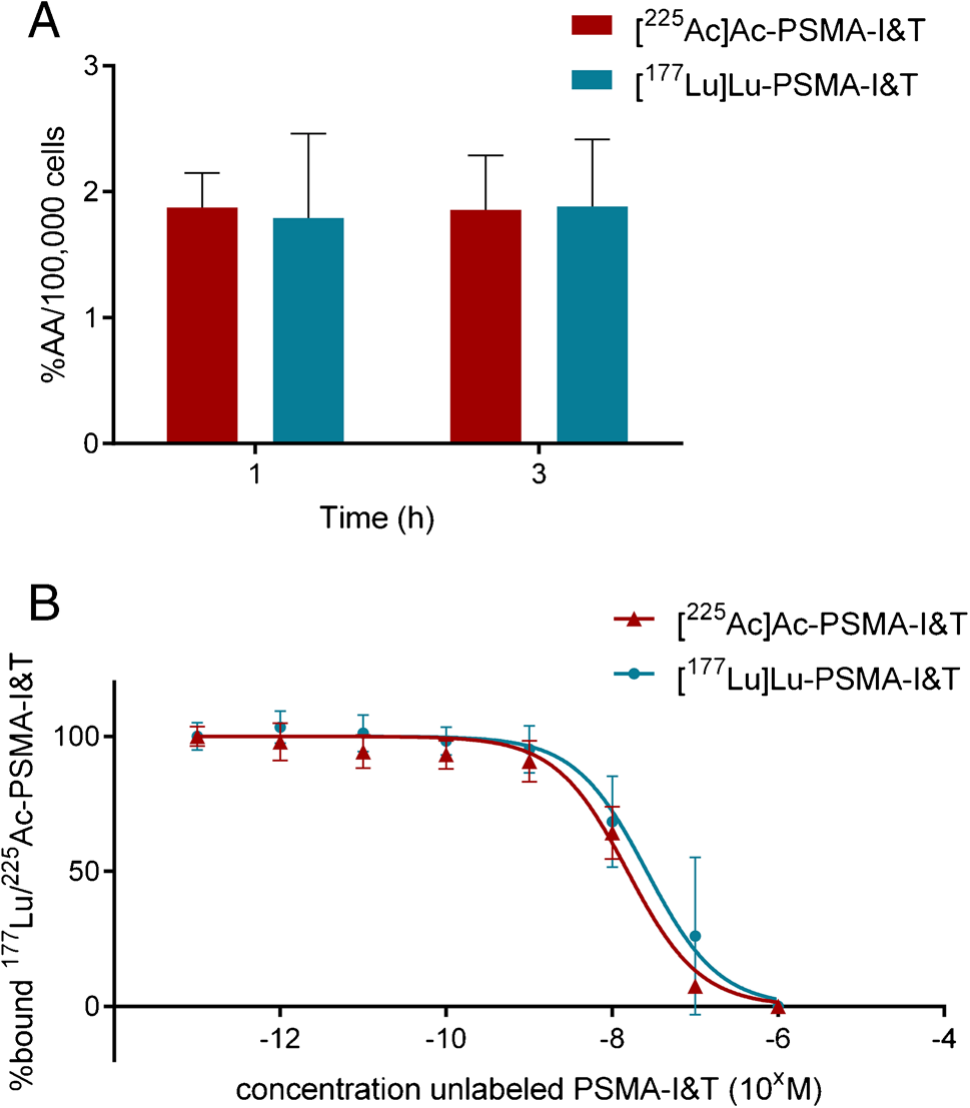
In vitro binding characteristics of [^225^Ac]Ac-PSMA-I&T and [^177^Lu]Lu-PSMA-I&T. **A** Uptake of [^225^Ac]Ac-PSMA-I&T and [^177^Lu]Lu-PSMA-I&T (both 0.225 MBq/nmol) after 1h and 3h of incubation (*n*=3). Uptake is expressed as the percentage added activity per 100,000 cells (%AA/100,000 cells). **B** IC_50_ curves of [^225^Ac]Ac-PSMA-I&T (0.225 MBq/nmol) and [^177^Lu]Lu-PSMA-I&T (40 MBq/nmol) assays at 1h of incubation (*n*=3). Error bars indicate standard deviation

In vitro uptake and displacement assays were performed in the PSMA positive PC3-PIP cell line to compare binding characteristics of [^225^Ac]Ac-PSMA-I&T and [^177^Lu]Lu-PSMA-I&T. These uptake experiments revealed the same uptake of [^225^Ac]Ac-PSMA-I&T and [^177^Lu]Lu-PSMA-I&T after 1 h (1.87± 0.28 and 1.79± 0.67 %AA/100,000 cells respectively) and 3 h of incubation (1.86± 0.43 and 1.88±0.53 %AA/100,000 cells respectively) (Figure 1A). IC_50_ displacement assays, during which increasing concentrations of unlabeled PSMA-I&T were used to block the radiolabeled PSMA-I&T, resulted in similar IC_50_ values for [^225^Ac]Ac-PSMA-I&T and [^177^Lu]Lu-PSMA-I&T in the nanomolar range (1.53E-08 M and 2.61E-08 M, respectively) (Figure 1B).

### [^225^Ac]Ac-PSMA-I&T-treated cells show slower DSB repair kinetics compared to [^177^Lu]Lu-PSMA-I&T-treated cells

**Fig. 2:**
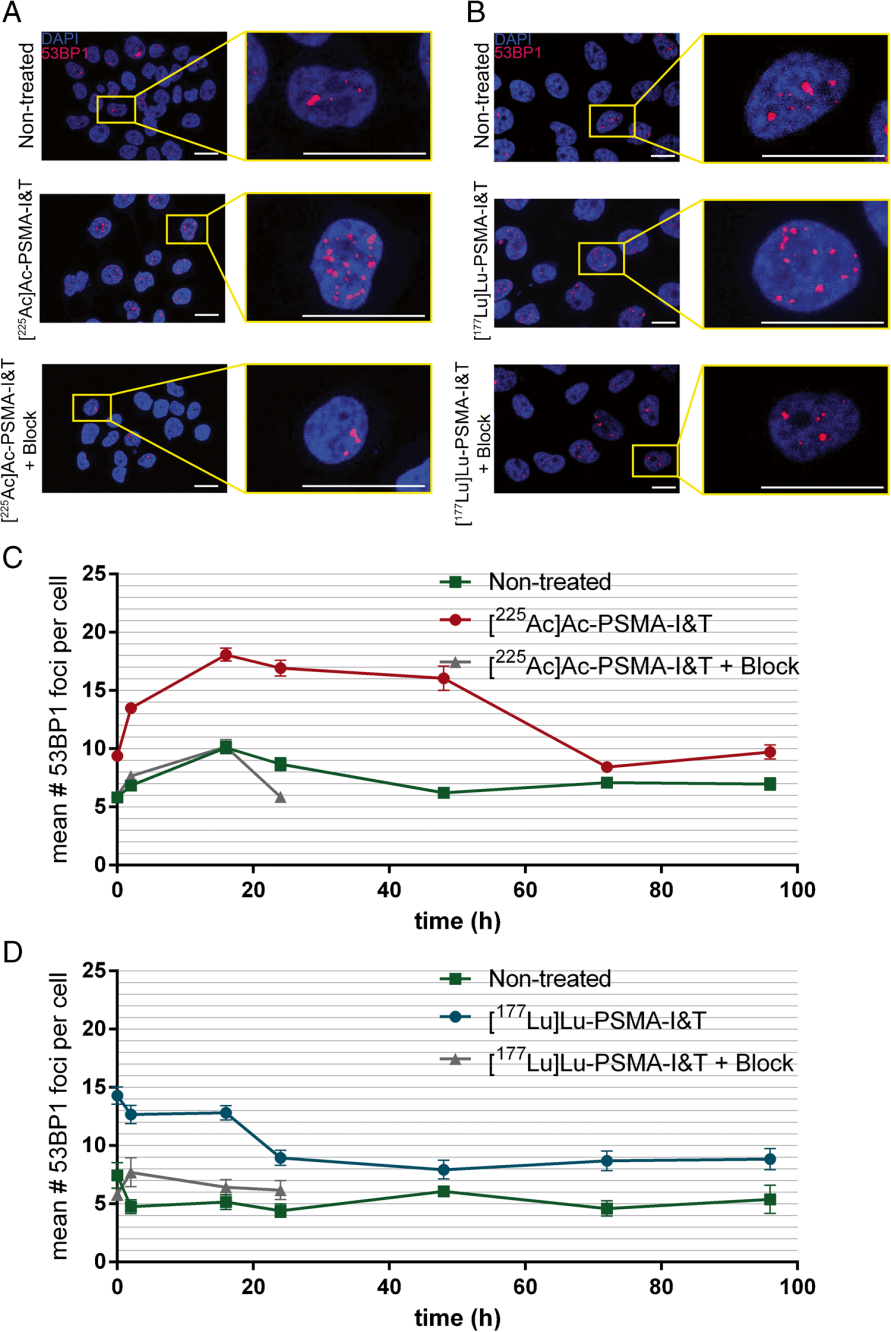
DSB analysis of cells treated with [^225^Ac]Ac-PSMA-I&T or [^177^Lu]Lu-PSMA-I&T based on 53BP1 foci quantification. **A** Representative images of cells 24 h after incubation with [^225^Ac]Ac-PSMA-I&T (0.37 kBq/mL with and without a block of ×1000 excess of unlabeled PSMA-I&T) and non-treated cells (scale bar = 10 μm). Blue: DAPI; red: 53BP1. **B** Representative images of PC3-PIP cells 24 h after incubation with [^177^Lu]Lu-PSMA-I&T (0.4 MBq/mL with and without block) and NT cells (scale bar = 10 μm). Blue: DAPI; red: 53BP1. **C** Representative graph of the average number of 53BP1 foci per nucleus of cells treated with 0.37 kBq/mL [^225^Ac]Ac-PSMA-I&T, non-treated cells, and cells treated with 0.37 kBq/mL [^225^Ac]Ac-PSMA-I&T with a block on different time points after incubation. **D** Representative graph of the average number of 53BP1 foci per nucleus of cells treated with 0.4 MBq/mL [^177^Lu]Lu-PSMA-I&T, non-treated cells, and cells treated with 0.4 MBq/mL [^177^Lu]Lu-PSMA-I&T with a block on different time points after incubation. Error bars indicate SEM. Experiment was performed twice; 1 representative graph for both conditions is presented here and the additional graphs can be found in the supplemental material (Figure S1)

**Fig. 3:**
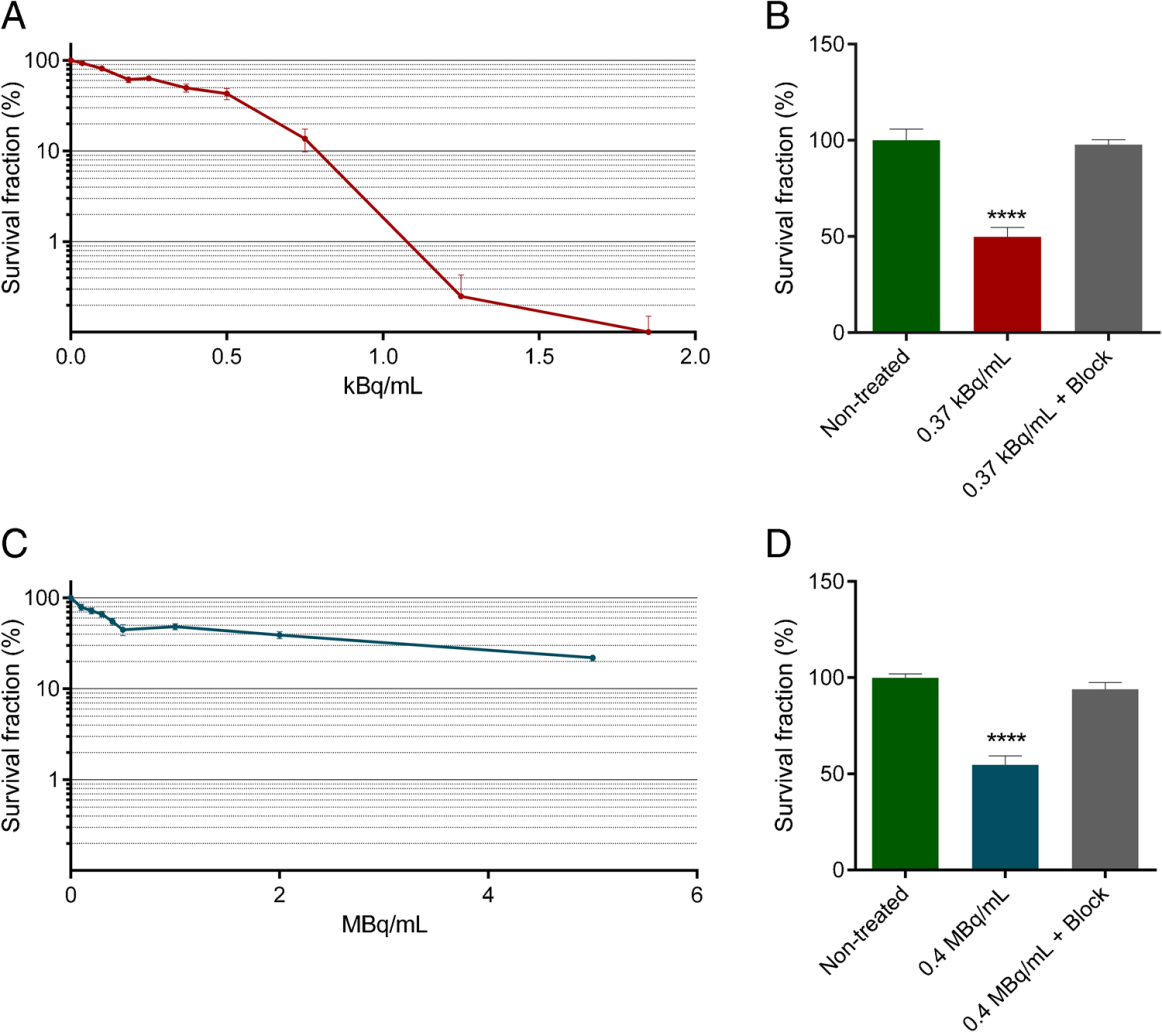
Cell killing efficacy of [^225^Ac]Ac-PSMA-I&T and [^177^Lu]Lu-PSMA-I&T. **A** Clonogenic survival of cells treated with increasing concentrations of [^225^Ac]Ac-PSMA-I&T for 3h. **B** Clonogenic survival of non-treated cells or cells treated with 0.37 kBq/mL [^225^Ac]Ac-PSMA-I&T, with or without ×1000 excess of unlabeled PSMA-I&T (block). **C** Clonogenic survival of cells treated with increasing concentrations of [^177^Lu]Lu-PSMA-I&T for 3h. **D** Clonogenic survival of non-treated cells or cells treated with 0.4 MBq/mL [^177^Lu]Lu-PSMA-I&T, with or without ×1000 excess of unlabeled PSMA-I&T (block). For all graphs, experiments were repeated three times. Error bars represent standard error of the mean (SEM). Asterisks indicate significance compared to non-treated (**** *p* ≤ 0.0001)

To determine DSB induction and repair kinetics, the number of 53BP1 foci was determined after treatment with 0.37 kBq/mL [^225^Ac]Ac-PSMA-I&T or 0.4 MBq/mL [^177^Lu]Lu-PSMA-I&T over time which are the concentrations at which the treated cells showed an clonogenic survival of ±50% (Figure 3). Cells treated with either [^225^Ac]Ac-PSMA-I&T or [^177^Lu]Lu-PSMA-I&T had a 2-fold increase in number of 53BP1 foci/cell directly after removal of the radiotracers compared to non-treated cells and cells receiving ×1000 excess of unlabeled PSMA-I&T (Figure 2, Figure S1). Cells treated with [^225^Ac]Ac-PSMA-I&T reached the peak mean number of 18.1±7.4 53BP1 foci per cell at 16 h after incubation while [^177^Lu]Lu-PSMA-I&T-treated cells showed the highest average amount of 53BP1 foci per cell, 14.3±6.4, directly after incubation. Furthermore, the number of 53BP1 foci of [^225^Ac]Ac-PSMA-I&T-treated cells remained augmented until 72h after incubation, while the number of 53BP1 foci of [^177^Lu]Lu-PSMA-I&T-treated cells decreased to control levels within 24 h after treatment (*p<*0.0001 between 53BP1 foci numbers of [^225^Ac]Ac-PSMA-I&T and [^177^Lu]Lu-PSMA-I&T-treated cells between 24 and 72h) (Figure 2, Figure S1).

### Specific and activity concentration dependent cell killing by [^225^Ac]Ac-PSMA-I&T and [^177^Lu]Lu-PSMA-I&T

Clonogenic assays were performed to assess cell survival after treatment with [^225^Ac]Ac-PSMA-I&T and [^177^Lu]Lu-PSMA-I&T. Figure 3A depicts the decrease in the survival for [^225^Ac]Ac-PSMA-I&T with increasing concentrations of radioactivity, with complete cell killing reached at a concentration of 1.85 kBq/mL. Treatment with a concentration of 0.37 kBq/mL led to ±50% survival. This decrease in survival was prevented by adding a ×1000 excess of unlabeled PSMA-I&T (Figure 3B). Likewise, increasing concentrations of [^177^Lu]Lu-PSMA-I&T led to a decrease in cellular survival, with the highest used concentration of 5 MBq/mL resulting in a survival fraction of 20% (Figure 3C). The same as in [^225^Ac]Ac-PSMA-I&T-treated cells, the ±50% decrease in survival caused by 0.4 MBq/mL [^177^Lu]Lu-PSMA-I&T could be blocked by a ×1000 increased concentration of unlabeled PSMA-I&T (Figure 3D).

### [^225^Ac]Ac-PSMA-I&T has a higher biological cell killing effectiveness compared to [^177^Lu]Lu-PSMA-I&T

In order to calculate the absorbed dose to the DNA during the survival experiments, the absorbed dose rate per unit activity (*S* values) was determined. The self-absorbed dose rate *S* values for actinium-225 (including all daughters) and lutetium-177 distributed either in the cell membrane or in the cytoplasm, accounting for the observed cellular range of dimensions (minimum, average, and maximum cell dimensions), are reported in Table 2. The *S* values for actinium-225 are 200-550-fold higher than the *S* values for lutetium-177, depending on source localization and cellular dimension. Furthermore, assuming the radionuclide localized on the cellular membrane rather than internalized reduces the absorbed dose delivered to the nucleus during the incubation phase (i.e. spherical shape approximation). The cross absorbed dose rate *S* value in the uptake phase for lutetium-177 is 1.13E-06 Gy(Bq*s)^-1^, irrespectively of the source localization.

**Table 2:** Self-absorbed dose rates to the nucleus per unit activity (Gy(Bq*s)^-1^) depending on radionuclide localization (i.e. cell membrane and cytoplasm) and cellular dimension (i.e. minimum, average and maximum)

*Floating set-up (spherical cells)*					
Dimension	Source compartment				
	Cell membrane			Cytoplasm	
	Lutetium-177	Actinium-225		Lutetium-177	Actinium-225
Minimum	2.02E-04	1.05E-01		3.67E-04	1.78E-01
Average	1.04E-04	5.63E-02		1.98E-04	1.01E-01
Maximum	6.40E-05	3.56E-02		1.23E-04	6.48E-02
*Attached cells set-up (ellipsoidal shape)*					
Dimension	Source compartment				
	Cell membrane			Cytoplasm	
	Lutetium-177	Actinium-225		Lutetium-177	Actinium-225
Minimum	3.43E-04	1.61E-01		2.14E-04	1.09E-01
Average	1.65E-04	8.31E-02		1.16E-04	6.15E-02
Maximum	7.66E-05	4.05E-02		5.89E-05	3.23E-02
*Contribution of the radioactive medium*					
	Lutetium-177		Actinium-225		
	2.30E-11		4.57E-09		

**Fig. 4:**
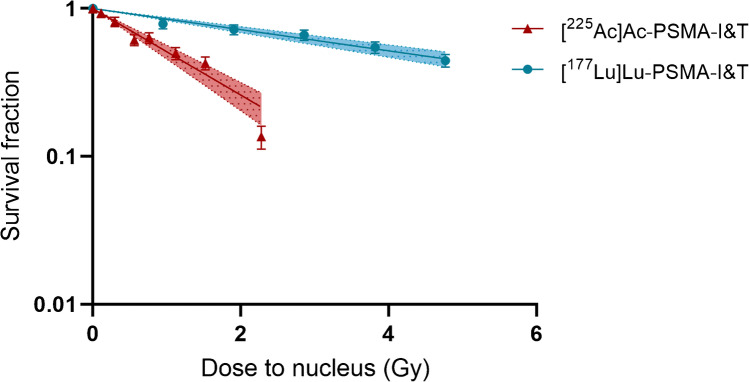
Dose-response curves of [^225^Ac]Ac-PSMA-I&T and [^177^Lu]Lu-PSMA-I&T

Next, the cellular excretion data indicated a biological half-life of radiolabeled PSMA-I&T (based on the cell binding of [^177^Lu]Lu-PSMA-I&T) of 2.3h plateauing at 41% of the initial bound activity (Figure S2). Combining the physical decay of each radionuclide with the biological time-activity curve described above and integrating over time, the time-integrated activity (Bq*s) after incubation was determined. Next, the absorbed dose was evaluated by combining *S* values and time-integrated activity calculations according to the MIRD scheme (Table 3). The contribution of the radioactive medium to the total absorbed dose during the incubation time was 1.6% and 2.6% for [^225^Ac]Ac-PSMA-I&T and [^177^Lu]Lu-PSMA-I&T, respectively. The absorbed dose corresponding to average sized cells was then correlated to the clonogenic assay results. Dose response curves according to the linear model were fitted with high correlation coefficients (*R*^2^> 0.96) for both [^225^Ac]Ac-PSMA-I&T and [^177^Lu]Lu-PSMA-I&T (Figure 4). The slope of the dose-response curves corresponds to the radiosensitivity of the cells to the treatment (also called radiosensitivity parameter or *α* [32]). *α* was 0.16±0.01 Gy^-1^ for [^177^Lu]Lu-PSMA-I&T and 0.67± 0.06 Gy^-1^ for [^225^Ac]Ac-PSMA-I&T, indicating a relative biological effectiveness (RBE) for cell killing of 4.2±0.46.

**Table 3:** Absorbed dose (Gy) accumulated over 7 days (i.e. duration of clonogenic survival assay) of either [^177L^u]Lu-PSMA-I&T or [^225^Ac]Ac-PSMA-I&T treatment. The absorbed dose is reported for 3 cellular dimension assumptions (i.e. average, minimum, and maximum) in order to provide the average and maximum range of variation of the absorbed dose calculations.

*[*^*177*^*Lu-PSMA-I&T*			
		Dimension	
Concentration (MBq/mL)	Average	Minimum	Maximum
0	0	0	0
0.1	0.74	1.48	0.37
0.2	1.48	2.96	0.74
0.3	2.22	4.43	1.11
0.4	2.96	5.91	1.48
0.5	3.70	7.39	1.85
*[*^*225*^*Ac]Ac-PSMA-I&T*			
		Dimension	
Concentration (kBq/mL)	Average	Minimum	Maximum
0	0	0	0
0.037	0.08	0.16	0.04
0.1	0.22	0.42	0.11
0.185	0.41	0.78	0.21
0.25	0.56	1.05	0.29
0.37	0.83	1.55	0.42
0.5	1.12	2.10	0.57
0.75	1.67	3.15	0.86

## Discussion

PSMA-TAT using actinium-225 is a novel, potent therapy option for patients with metastasized CRPC, which is potentially more effective compared to PSMA-RTL using lutetium-177. To further aid clinicians to choose the most optimal therapy for their PCa patients, a deeper understanding about the biological effects of PSMA-TAT, and more knowledge about the potential differences between e.g. lutetium-177-labeled and actinium-225-labeled PSMA-tracers, is indispensable. To this end, we performed a preclinical comparison of actinium-225- and lutetium-177-labeled PSMA-I&T, and report on important characteristics, such as cell binding, uptake, and dosimetry as well as biological effects in vitro.

We showed that [^225^Ac]Ac-PSMA-I&T and [^177^Lu]Lu-PSMA-I&T have the same uptake and binding affinity during in vitro assays, indicating that the type of radionuclide does not influence the binding characteristics and showing that tracer binding did not had an impact on the biological effects that were observed. Similarly, previous research has shown that [^177^Lu]Lu-PSMA-617 and [^161^Tb]Tb-PSMA-617 have a similar pharmacokinetic profile in vitro and in vivo [4, 33]. Likewise, comparable pharmacokinetics have been shown in vitro between [^68^Ga]Ga-PSMA-617, [^44^Sc]Sc-PSMA-617, and [^177^Lu]Lu-PSMA-617 [34]. Albeit similar in vitro binding between [^225^Ac]Ac-PSMA-I&T and [^177^Lu]Lu-PSMA-I&T allows us to make fair radiobiological comparisons, further in vivo and clinical studies are necessary to investigate the potential differences in biodistribution, and thus tumor uptake, of the two radionuclides, which may impact their effectiveness. This is important as previous studies have shown that a change of radionuclide could potentially influence the biodistribution of different radiopharmaceuticals in mice [34, 35]. Moreover, because of the different molar activities for [^225^Ac]Ac-PSMA-I&T 0.225 MBq/nmol) and [^177^Lu]Lu-PSMA-I&T (40 MBq/nmol), and the increasing concentrations we used, the total added mass PSMA-I&T differed between the conditions. However, because the used molarities remained in the nanomolar range (0.2×10^-9^ M − 1.25×10^-8^ M), these differences in mass were assumed to not influence therapeutic outcome.

In this study, 0.37 kBq/mL of [^225^Ac]Ac-PSMA-I&T and 0.4 MBq/mL of [^177^Lu]Lu-PSMA-I&T resulted in similar survival of the cells. By performing macrodosimetry on these data, we calculated a 4.2 higher in vitro RBE for [^225^Ac]Ac-PSMA-I&T as compared to [^177^Lu]Lu-PSMA-I&T. The lack of detailed in vitro literature on the comparison between actinium-225- and lutetium-177-labeled radiopharmaceuticals limits direct RBE comparisons with other studies. However, the RBE of 4.2 in the benefit of [^225^Ac]Ac-PSMA-I&T found here is in line with the general assumed RBE of 3-5 for *α*-emitters compared to β-particle radiation [36].

Even though our RBE calculations are in line with literature, we made various assumptions in the dose-response modeling which could influence the estimation of the overall therapeutic efficacy. For our calculations, we have used the commonly used macrodosimetric method. However, the short path length of *α*-emitters and the possible inhomogeneous activity distribution over the cell population may require the use of a microdosimetric approach. Future studies using a microdosimetric approach evaluating the absorbed specific energy deposited to the target regions, including the stochastic effect at cellular level, could contribute to a better prediction of this therapeutic effect [37]. Cell proliferation, oxygenation, and dose rate are well-known factors that can influence the biological response to low LET radiation (such as β-emissions from lutetium-177) and should therefore theoretically be included in dose-response modeling [38, 39]. However, since the influence of these factors has not been elucidated yet for RLT, and high LET radiation in specific is known not to be influenced by these factors, they were not included in the dose-response modeling in this study [39]. Furthermore, we must note that dosimetry was based on uptake data performed with adherent cells, while during the clonogenic assay, the cells were treated in suspension. This might have led to an underestimation of the absorbed dose, as cells in suspension might have larger membrane surface exposed to the radioactive medium.

The dose calculations accounting for the biological excretion in the medium showed that only 2–3% of the cumulative absorbed dose is delivered by the radioactive medium. This low percentage of cumulative absorbed dose is in line with our findings in the survival and DSB assays, demonstrating that adding a block to the radioactive medium (×1000 excess of unlabeled PSMA-I&T) completely abrogates the therapeutic effects. Similarly, in previously published studies, no decrease in cellular survival was observed after treatment with lutetium-177-labeled diethylenetriaminepentaacetic acid (DTPA) used as a negative control since it is unable to bind to the cells [33, 40].

The higher RBE of [^225^Ac]Ac-PSMA-I&T explains the increased toxicity of [^225^Ac]Ac-PSMA-I&T over [^177^Lu]Lu-PSMA-I&T during the survival assay. Moreover, it might also explain the difference in DSBs induction and repair kinetics as observed for [^225^Ac]Ac-PSMA-I&T with higher number of DSBs after 24h of incubation as compared to [^177^Lu]Lu-PSMA-I&T, while both used concentrations led to a similar survival in the clonogenic assay. The different DSB repair kinetics observed for actinium-225- and lutetium-177-treated cells could be caused by differences in the type of induced DSBs, as the emitted *α*-particles of actinium-225 most likely induce more complex and harder to repair DSBs compared to lutetium-177, leading to a persistent level of DSBs in the actinium-225-treated cells. Indeed, it was previously shown that there is a difference in the induction and repair of DSBs after *α*-particle irradiation compared to X-ray radiation, which has a low LET comparable to that of lutetium-177 [7]. In this study, cells irradiated with an external source of alpha-particle emitter americium-241 showed higher numbers of DSB, and resolving of the DSBs was slower compared to X-ray induced DSBs. Although this research was conducted using external radiation and not RLT, these results are in line with our observation that DSBs induced by [^225^Ac]Ac-PSMA-I&T have a different induction and repair kinetics compared to [^177^Lu]Lu-PSMA-I&T-induced DSBs.

The higher RBE of [^225^Ac]Ac-PSMA-I&T over [^177^Lu]Lu-PSMA-I&T is most probably also causing the major downside of PSMA-TAT, namely increased radiotoxicity. Future in vivo and in vitro studies focusing on decreasing the impact of PSMA-TAT to such PSMA-expressing organs like the salivary glands while maintaining its therapeutic efficacy are of great importance.

The current study was conducted in a simplified model of prostate cancer that does not fully recapitulate the complexity of clinical prostate cancer. Therefore, the in vitro RBE might not fully represent the RBE in patients, as e.g. tumor size and tumor microenvironment (including vasculature, stromal, and immune cells) can influence the effects of PSMA-RLT and PSMA-TAT. Therefore, follow-up studies using models with heterogeneous PSMA expression, such as patient-derived (primary) organoid cultures and xenograft models with endogenous PSMA expression, that more closely recapitulate clinic, are essential to fully compare the effects of actinium-225- and lutetium-177-labeled PSMA tracers.

To conclude, [^225^Ac]Ac-PSMA-I&T has a 4.2 times higher in vitro therapeutic efficacy compared to [^177^Lu]Lu-PSMA-I&T. [^225^Ac]Ac-PSMA-I&T induces a higher number, and most likely more complex DSBs, compared to [^177^Lu]Lu-PSMA-I&T which could explain this higher RBE. Altogether, our results could contribute to rational design of PSMA-RLT regimens when decisions between (dosing of) actinium-225 and lutetium-177 have to be made.

## Supplementary Information

ESM 1 (DOCX 33 kb)

Supplemental Figure 1 Additional DNA double strand break analysis on PC3-PIP cells treated with [^225^Ac]Ac-PSMA-I&T or [^177^Lu]Lu-PSMA-I&T based on 53BP1 foci. (A) Average amount of 53BP1 foci per nucleus of cells treated with 0.37 kBq/mL [^225^Ac]Ac-PSMA-I&T, non-treated cells and cells treated with 0.37 kBq/mL [^225^Ac]Ac-PSMA-I&T with a block on different time points after incubation. (B) Average amount of 53BP1 foci per nucleus of cells treated with 0.4 MBq/mL [^177^Lu]Lu-PSMA-I&T, non-treated cells and cells treated with 0.4 MBq/mL [^177^Lu]Lu-PSMA-I&T with a block on different time points after incubation. Error bars indicate SEM. (JPG 6032 kb)

Supplemental Figure 2 Single exponential plotted excretion rate of [^177^Lu]Lu-PSMA-I&T (40 MBq/nmol, 10E-09M) treated cells after 3h incubation. Error bars indicate standard deviation and the shaded area indicates the 95% confidence interval of the fit. (PNG 49 kb)

## Data Availability

Please contact the corresponding author.
