# Correction to: In vitro dose effect relationships of actinium‑225‑ and lutetium‑177‑labeled PSMA‑I&T

**DOI:** 10.1007/s00259-022-05845-2

**Published:** 2022-05-26

**Authors:** Eline A. M. Ruigrok, Giulia Tamborino, Erik de Blois, Stefan J. Roobol, Nicole Verkaik, Marijke De Saint-Hubert, Mark W. Konijnenberg, Wytske M. van Weerden, Marion de Jong, Julie Nonnekens

**Affiliations:** 1 Department of Radiology and Nuclear Medicine, Erasmus MC Cancer Institute, Erasmus University Medical Center, 3000 CA Rotterdam, The Netherlands; 2 Department of Experimental Urology, Erasmus University Medical Center, 3000 CA Rotterdam, The Netherlands; 3 Research in Dosimetric Application, Belgian Nuclear Research Centre (SCK CEN), Mol, Belgium; 4 Department of Molecular Genetics, Erasmus MC Cancer Institute, Erasmus University Medical Center, 3000 CA Rotterdam, The Netherlands

**Correction to: European Journal of Nuclear Medicine and Molecular Imaging**

10.1007/s00259-022-05821-w

The publisher regrets that the Tables 2 and 3 that appear in the original article are incorrect. The correct tables appear below.

**Table 2:** Self-absorbed dose rates to the nucleus per unit activity (Gy(Bq*s)^-1^) depending on radionuclide localization (i.e. cell membrane and cytoplasm) and cellular dimension (i.e. minimum, average and maximum)

*Floating set-up (spherical cells)*				
Dimension	Source compartment			
	Cell membrane		Cytoplasm	
	Lutetium-177	Actinium-225	Lutetium-177	Actinium-225
Minimum	2.02E-04	1.05E-01	3.67E-04	1.78E-01
Average	1.04E-04	5.63E-02	1.98E-04	1.01E-01
Maximum	6.40E-05	3.56E-02	1.23E-04	6.48E-02
*Attached cells set-up (ellipsoidal shape)*				
Dimension	Source compartment			
	Cell membrane		Cytoplasm	
	Lutetium-177	Actinium-225	Lutetium-177	Actinium-225
Minimum	3.43E-04	1.61E-01	2.14E-04	1.09E-01
Average	1.65E-04	8.31E-02	1.16E-04	6.15E-02
Maximum	7.66E-05	4.05E-02	5.89E-05	3.23E-02
*Contribution of the radioactive medium*				
	Lutetium-177		Actinium-225	
	2.30E-11		4.57E-09

**Table 3:** Absorbed dose (Gy) accumulated over 7 days (i.e. duration of clonogenic survival assay) of either [^177L^u]Lu-PSMA-I&T or [^225^Ac]Ac-PSMA-I&T treatment. The absorbed dose is reported for 3 cellular dimension assumptions (i.e. average, minimum and maximum) in order to provide the average and maximum range of variation of the absorbed dose calculations

*[*^*177*^*Lu]Lu-PSMA-I&T*			
		Dimension	
Concentration (MBq/mL)	Average	Minimum	Maximum
0	0	0	0
0.1	0.74	1.48	0.37
0.2	1.48	2.96	0.74
0.3	2.22	4.43	1.11
0.4	2.96	5.91	1.48
0.5	3.70	7.39	1.85
*[*^*225*^*Ac]Ac-PSMA-I&T*			
		Dimension	
Concentration (kBq/mL)	Average	Minimum	Maximum
0	0	0	0
0.037	0.08	0.16	0.04
0.1	0.22	0.42	0.11
0.185	0.41	0.78	0.21
0.25	0.56	1.05	0.29
0.37	0.83	1.55	0.42
0.5	1.12	2.10	0.57
0.75	1.67	3.15	0.86

The original article has been corrected.

