# Supplementary material for: In vitro dose effect relationships of actinium-225- and lutetium-177-labeled PSMA-I&T
Source: Eur J Nucl Med Mol Imaging. 2022 May 12;49(11):3627–38. doi: 10.1007/s00259-022-05821-w (PMC9399067; doi:10.1007/s00259-022-05821-w)
Supplement: Supplementary file 1 — (DOCX 33 kb) [file 259_2022_5821_MOESM1_ESM.docx]

Supplemental material.

Material & Methods – Dosimetry

Dosimetry was performed to calculate the total absorbed dose to the nucleus of the cells during the clonogenic assay. We differentiated between the incubation period with the radioactive medium and the colony growth period. First, the cumulated activity in each “x” compartment ($\tilde{A\left( t \right)}_{x}$) was calculated, whereafter the absorbed dose was determined by multiplying ($\tilde{A\left( t \right)}_{x}$) to the corresponding S-value. Below, we described the calculations in more detail.

**Incubation period (Time: 0 - 3 h):**

Cumulated activity in cells:

$${\tilde{A\left( t \right)}_{CS}=0.76 x A}_{0}f_{B}\int_{0}^{3h} e^{(-\lambda_{p}t)} dt$$

$${\tilde{A\left( t \right)}_{Cy}=(1-0.76) x A}_{0}f_{B}\int_{0}^{3h} e^{(-\lambda_{p}t)} dt$$

$${\tilde{A\left( t \right)}_{M}= A}_{0}\int_{0}^{3h} e^{(-\lambda_{p}t)} dt$$

With:

*CS: Cell surface*

*Cy: Cytoplasm*

*M: Medium*

$f_{B}$*: Bound A fraction over the whole cell*

$A_{0}$ *: Added activity*

$\lambda_{p}$ *: Physical half-life (permanent bound assumption in the first 3h)*

*0.76: The fraction of bound activity assumed to be localized in the membrane.*

The absorbed dose was then evaluated by multiplying each term for the corresponding S-value reported in Table 2.

$D_{CS}$= $\tilde{A\left( t \right)}_{CS} x S_{N\leftarrow CS}$

$D_{Cy}$= $\tilde{A\left( t \right)}_{Cy} x S_{N\leftarrow Cy}$

$D_{M}$= $\tilde{A\left( t \right)}_{M} x S_{N\leftarrow M}$

**Colony growth period (Time: 3h – 7d):**

During the colony formation phase, the activity was not permanently bound to the cells but followed the curve shown in Supplemental material (Fig S2). The bound fraction of activity decreases as:

$${f_{B\_new}=f_{B} (e}^{(-\lambda_{b} t)}+P)$$

Where, fB is the bound fraction at the end of the incubtion time, as in the previous section, and P is the plateau level.

Incorporating this biological excretion factor with the physical decay:

$${\tilde{A\left( t \right)}_{CS}=0.76 A}_{0}f_{B}\int_{3h}^{7d} e^{-(\lambda_{b}+ \lambda_{p})t}+Pe^{( -\lambda_{p})t} dt$$

$${\tilde{A\left( t \right)}_{Cy}=(1-0.76) A}_{0} f_{B}\int_{3h}^{7d} e^{-(\lambda_{b}+ \lambda_{p})t}+Pe^{( -\lambda_{p})t} dt$$

The medium was considered free of radioactivity and hence it did not contribute to the dose absorbed to the nucleus. Hence:

$D_{CS}$= $\tilde{A\left( t \right)}_{CS} x S_{N\leftarrow CS}$

$D_{Cy}$= $\tilde{A\left( t \right)}_{Cy} x S_{N\leftarrow Cy}$
