# Supplementary figures and images for: In vitro dose effect relationships of actinium-225- and lutetium-177-labeled PSMA-I&T
Source: Eur J Nucl Med Mol Imaging. 2022 May 12;49(11):3627–38. doi: 10.1007/s00259-022-05821-w (PMC9399067; doi:10.1007/s00259-022-05821-w)

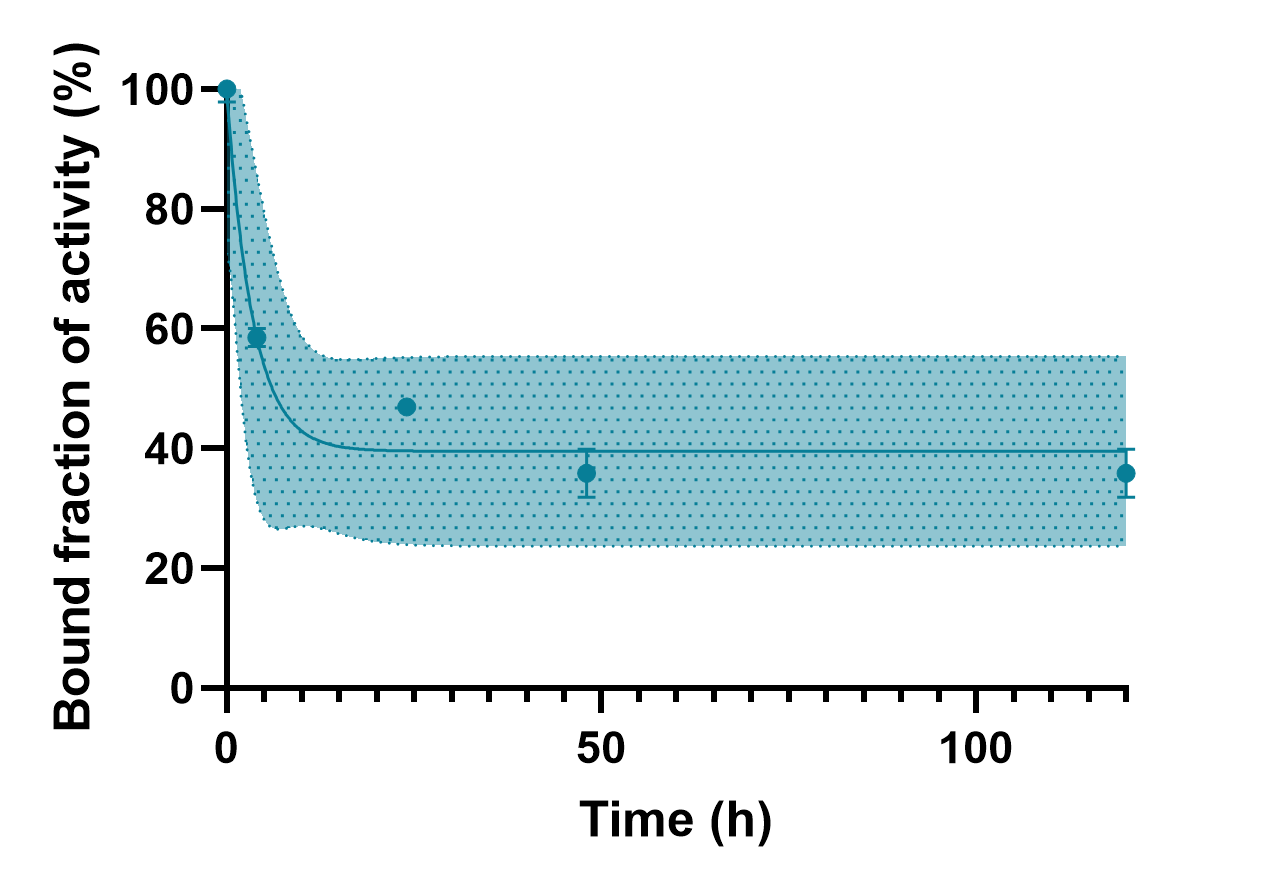

Supplement: Supplementary file 3 — Single exponential plotted excretion rate of [177Lu]Lu-PSMA-I&T (40 MBq/nmol, 10E-09M) treated cells after 3h incubation. Error bars indicate standard deviation and the shaded area indicates the 95% confidence interval of the fit. (PNG 49 kb) [file 259_2022_5821_MOESM3_ESM.png]
